# Supplementary material for: Long-Term Aging of Concentrated Aqueous Graphene Oxide Suspensions Seen by Rheology and Raman Spectroscopy
Source: Nanomaterials (Basel). 2022 Mar 10;12(6):916. doi: 10.3390/nano12060916 (PMC8950440; doi:10.3390/nano12060916)
Supplement: Supplementary file 1 [file nanomaterials-12-00916-s001.zip › nanomaterials-1585307-supplementary-proof done_corrected KL.pdf]

Supplementary Materias
Long-Term Aging of Concentrated Aqueous Graphene Oxide Suspensions Seen by Rheology and Raman Spectroscopy

Benjámin Gyarmati [1], Shereen Farah [1], Attila Farkas [2], György Sáfrán [3], Loredana Mirela Voelker-Pop [4] and Krisztina László [1,*]

[1] Department of Physical Chemistry and Materials Science, Faculty of Chemical Technology and Biotechnology, Budapest University of Technology and Economics, Műegyetem rkp. 3, H-1111 Budapest, Hungary; gyarmati.benjamin@vbk.bme.hu (B.G.); sfarah@edu.bme.hu (S.F.)

[2] Department of Organic Chemistry and Technology, Faculty of Chemical Technology and Biotechnology, Budapest University of Technology and Economics, Műegyetem rkp. 3, H-1111 Budapest, Hungary; farkas.attila@vbk.bme.hu

[3] Research Institute for Technical Physics and Materials Science, Eötvös Loránd Research Network, Konkoly Thege M. út 29-33, H-1121 Budapest, Hungary; safran.gyorgy@energia.mta.hu

[4] Anton Paar Germany GmbH, Helmuth-Hirth-Strasse 6, D-73760 Ostfildern, Germany; loredana.voelker-pop@anton-paar.com

* Correspondence: laszlo.krisztina@vbk.bme.hu; Tel.: +3614631893

Table S1. Rheological studies on high concentration aqueous GO suspensions.

Concentration Range		Additional Information about GO			Methods				References
					Rheology				
As Reported	*v*/*v*% converted[1]	Synthesis Reference	Density (g/cm^3)	Aspect Ratio	Steady Shear	Oscillatory Shear	Transient Shear	Characterization Techniques	
0.1-3 *v*/*v*%		[5, 33]	×	Lateral: 64 µm, thickness: a few nm	✓	✓	-	Optical microscopy; Raman spectroscopy; Elemental chemical analysis Surface tension	[3]
1-9 mg/mL	0.07-0.65	[11, 13]	×	1000-5000	✓	-	-	AFM; SEM; XRD	[22]
0.002-0.6 *v*/*v*% 0.05-13.3 mg/mL		[34, 35, 36]	×	45,000	✓	✓	-	AFM; SEM	[23]
0.004-3.5 *m*/*m*%	0.003-2.52	Commercial[2]	2.2 [37]	1160	✓	✓	✓	AFM Zeta potential	[26]
6-25 mg/mL	0.43-1.80	[18]	×	1000	✓	✓	✓	AFM; TEM	[27]
0.08-1.8 *v*/*v*%		[17]	×	700	✓	✓	-	AFM; SEM; Polarized optical microscopy	[38]

0.03-8 $v/v\%$		[16]	0.002	600	✓	✓	-	AFM	[39]
1-10 mg/mL	0.07–0.72	[14, 15, 40]	×	2500	✓	-	-	AFM; SEM;<br>Polarized optical microscopy	[41]
0.005-2.22 $v/v\%$<br>0.1-40 mg/mL		[11]	×		✓	-	-	AFM; SEM; XRD; FTIR; Polarized optical microscopy; Raman spectroscopy;<br>Surface tension; Contact angle	[42]
200 mg/mL	14	Commercial[3]	×	×	✓	✓	-	SEM; XRD<br>Raman spectroscopy; FTIR;<br>N_2 adsorption	[43]
0.72 $v/v\%$<br>10 mg/mL	0.72	[12]	1.39		✓	✓	✓	TEM; XRD; XPS; FTIR; SEM;<br>Polarized light imaging; Raman spectroscopy	[This work]

[1]For the purposes of comparison volume fractions were calculated using the density 1.39 g/cm^3 reported in this work.

[2] GO sheets from Graphenea, Spain

[3] GO powder from The Sixth Element (Changzhou) Materials Technology.

Table S2. Fitted parameters from the Bingham and power law models.

Model	Dataset in Fig. S3	τ_0 (Pa)	k (Pa·s)	n	R^2
Bingham	1	24 ± 1	0.75 ± 0.04	-	0.9143
	2	29 ± 1	0.84 ± 0.05	-	0.9152
	3	31 ± 2	0.89 ± 0.05	-	0.9089
Power-law	1	-	21 ± 1	0.29 ± 0.01	0.9757
	2	-	26 ± 1	0.27 ± 0.01	0.9715
	3	-	28 ± 1	0.27 ± 0.01	0.9757

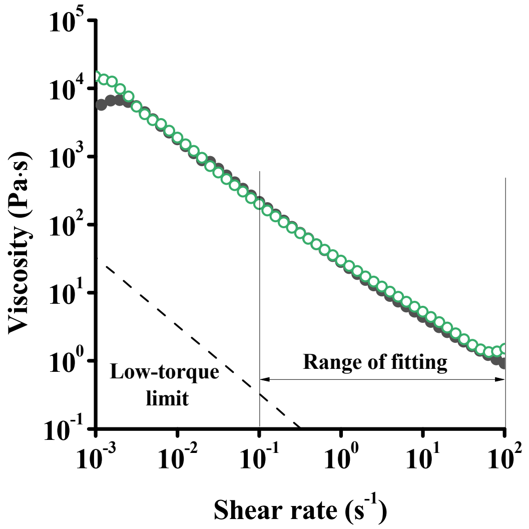

Figure S1. Effect of measurement time on the response curve. Black full symbol: 10 s/point (original measurement time); green open symbol: 100 s/point. In the shear rate range 0.001 to 100 s^{-1} 10 points per decade were recorded. The low-torque limit of viscosity calculated for 25 mm diameter parallel plate (PP25) used is indicated with dashed line. The minimum torque (0.1 μNm) of the instrument was used for the calculation.

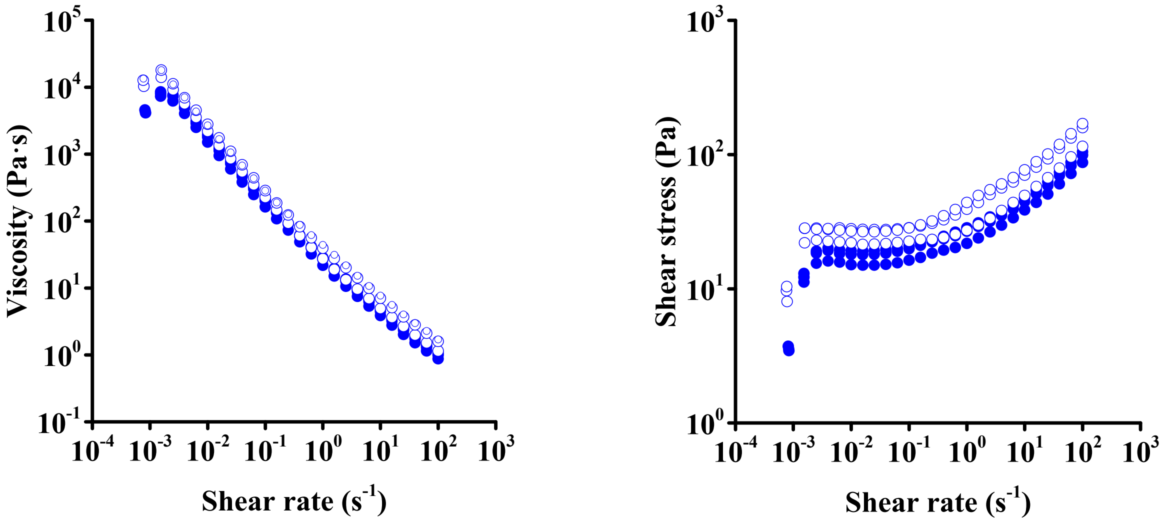

(A) (B)

Figure S2. Steady shear rate measurements on fresh (0 y) aqueous GO suspensions. Influence of shear rate on viscosity (**A**) and shear stress (**B**). Full and open symbols correspond to samples before and after the transient measurement, respectively.

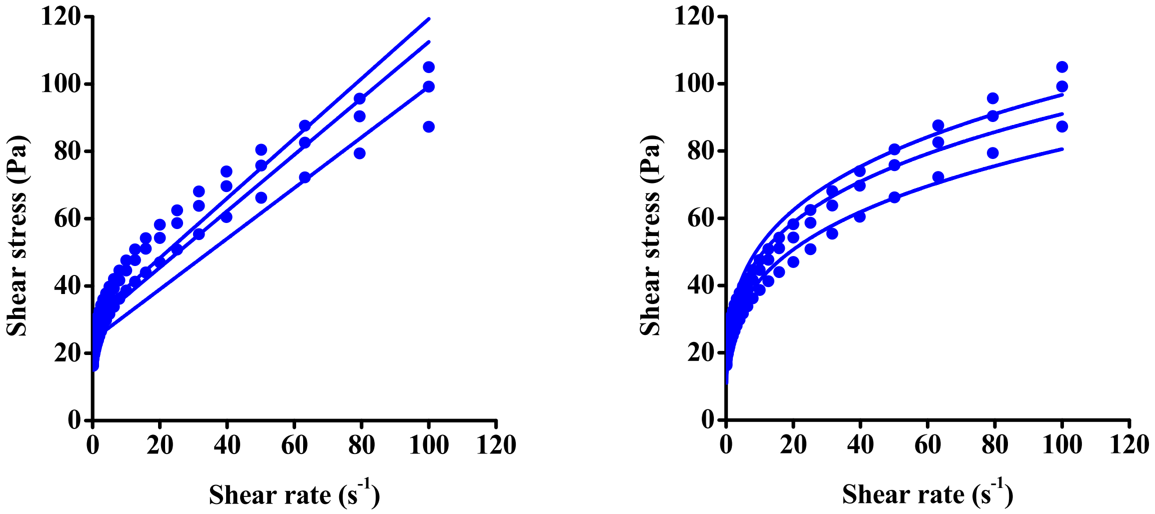

(A) (B)

Figure S3. Experimental shear stress – shear rate data obtained on fresh samples (symbols) and their fits to Bingham (**A**) and power law (**B**) models (lines).

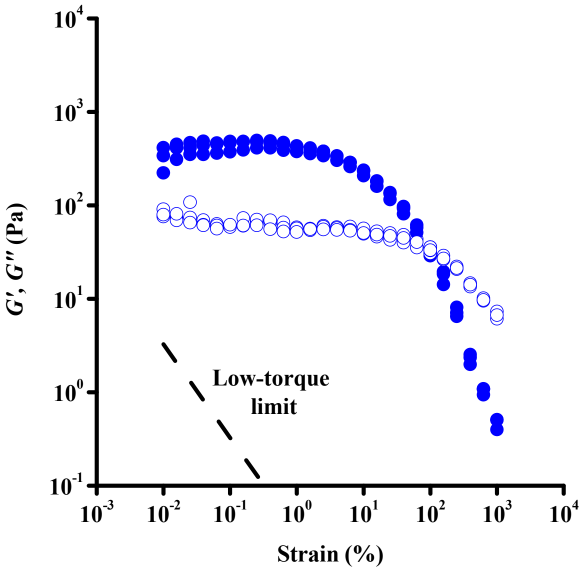

Figure S4. Results of strain-dependent oscillatory shear measurements on the fresh aqueous GO suspension at constant frequency 1 rad·s^{-1}. Solid and open symbols represent the storage and loss moduli, respectively. The low-torque limit of viscosity calculated for 25 mm diameter parallel plate (PP25) used is indicated with dashed line.
The minimum torque (0.1 μNm) of the instrument was used for the calculation.

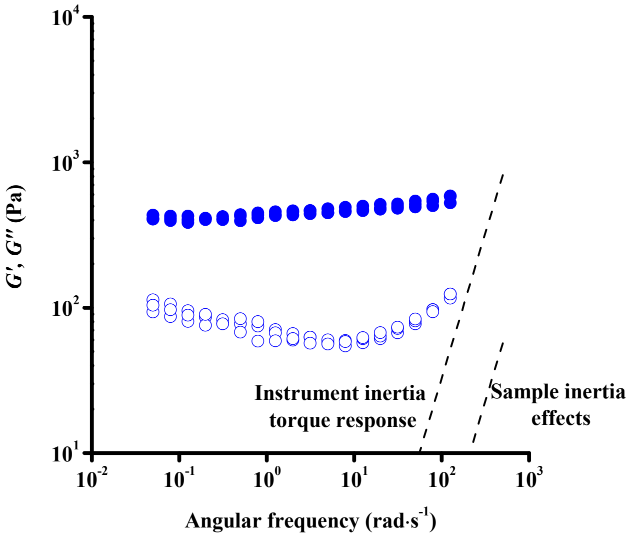

Figure S5. Dynamic frequency sweep of fresh (0 y) aqueous GO suspension at constant strain amplitude 0.1%. Solid and open symbols represent the storage and loss moduli, respectively. Sample inertia limit was estimated by comparing the wavelength of propagating waves in the material to gap size as proposed by Ewoldt et al. [52] The following values were used: $\cos^2(\delta/2) = 1$; ϱ = 1000 kg·m^{-3}; D = 0.3 mm. The inertia of the measuring system was measured at 100 Hz and torque response curve was calculated by estimating the precision of inertia correction (ε) to be 0.01.

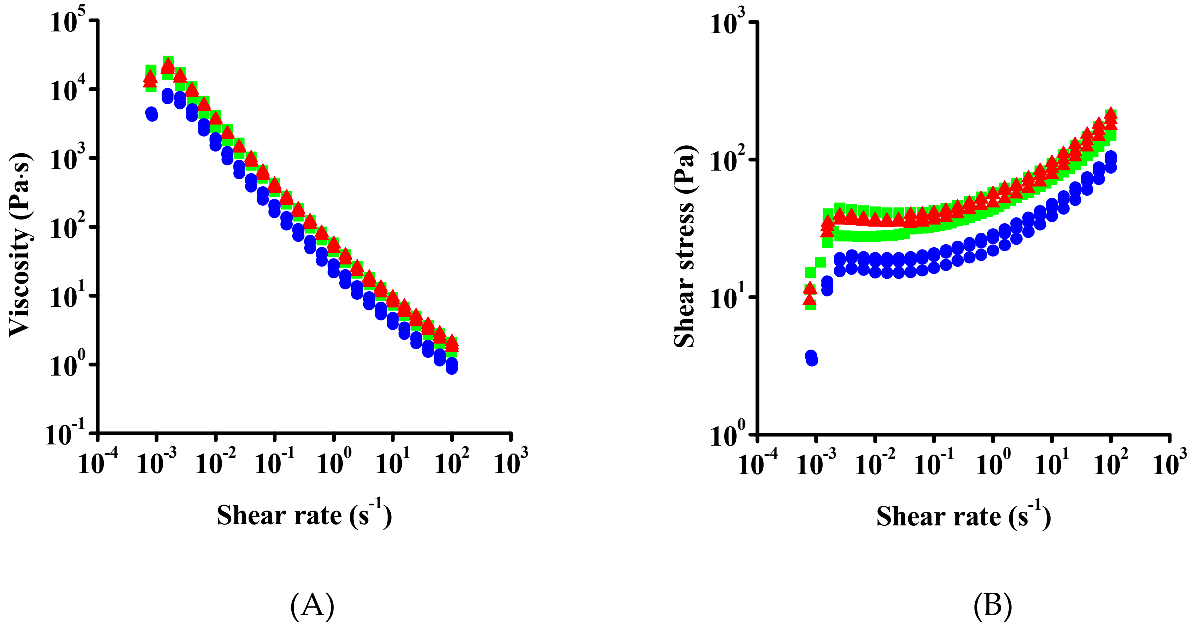

Figure S6. Steady shear measurements on aqueous GO suspensions of various ages. Influence of shear rate on viscosity (**A**) and shear stress (**B**). Blue dots: 0 y; red triangles 1 y; green squares: 2 y.

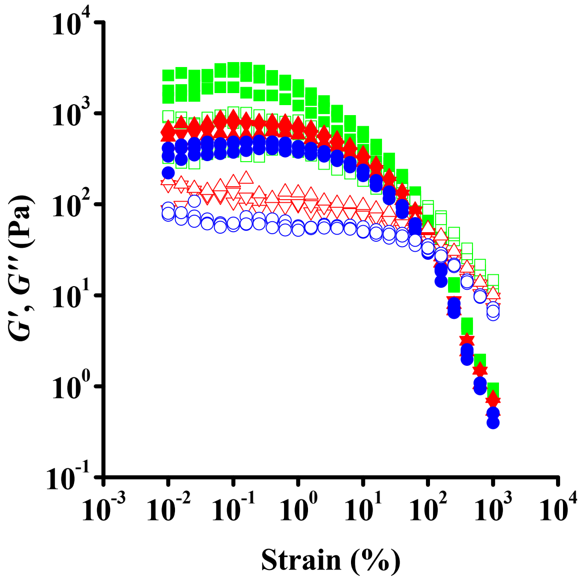

Figure S7. Results of strain-dependent oscillatory shear measurements on aqueous GO suspensions of various ages at constant frequency 1 rad·s^{-1}. Blue dots: 0 y; red triangles: 1 y; green squares: 2 y. Solid and open symbols represent the storage and loss moduli, respectively.

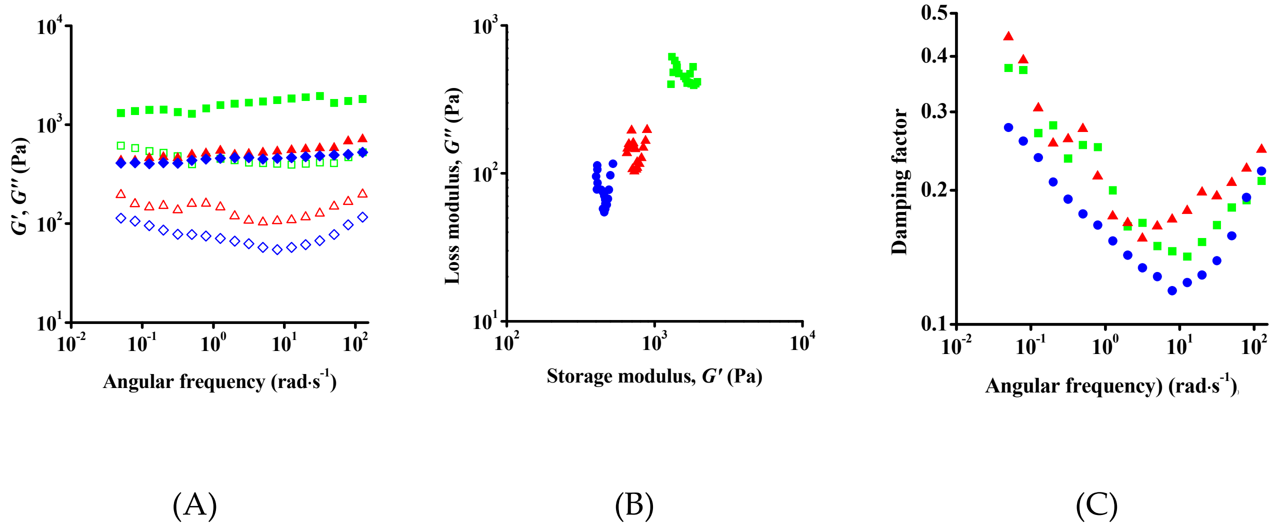

Figure S8. (**A**) Dynamic frequency sweep carried out within LVR at constant strain amplitude 0.1% on aqueous GO suspensions of different ages. Solid and open symbols represent the storage and loss moduli, respectively. (**B**) Cole-Cole representation of GO suspensions. (**C**) Frequency-dependence of damping factor. Blue dots: 0 y; red triangles: 1 y; green squares: 2 y.

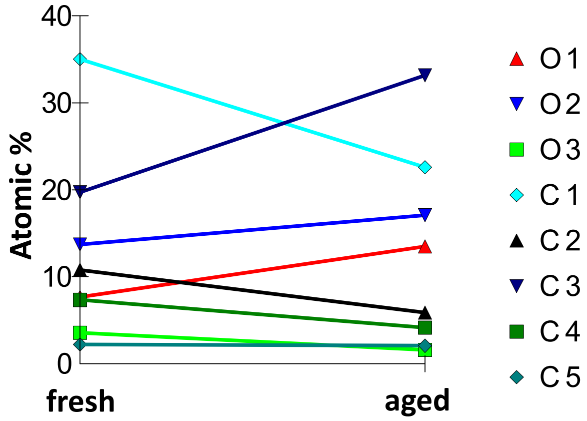

Figure S9 Change of the various O1s and C1s states in time. (Probes from the "2y" sample were freeze dried and analyzed when it was fresh and after 2 years.).
